# Culture-dependent screening of endospore-forming clostridia in infant feces

**DOI:** 10.1186/s12866-023-03104-4

**Published:** 2023-11-17

**Authors:** Eugenio Ingribelli, Nikol Modrackova, Vaclav Tejnecky, Jiri Killer, Clarissa Schwab, Vera Neuzil-Bunesova

**Affiliations:** 1 Department of Microbiology, Nutrition and Dietetics, Czech University of Life Sciences Prague, Prague, Czechia; 2 Department of Soil Science and Soil Protection, Czech University of Life Sciences Prague, Prague, Czechia; 3 Institute of Animal Physiology and Genetics v.v.i, the Czech Academy of Sciences, Prague, Czechia; 4 Biological and Chemical Engineering, Aarhus University, Aarhus C, Denmark

**Keywords:** Infant gut microbiota, Cultivation, Endospore formers, *Clostridium*, Fermentation profiles, Butyrate

## Abstract

**Background:**

Only a few studies dealt with the occurrence of endospore-forming clostridia in the microbiota of infants without obvious health complications.

**Methods:**

A methodology pipeline was developed to determine the occurrence of endospore formers in infant feces. Twenty-four fecal samples (FS) were collected from one infant in monthly intervals and were subjected to variable chemical and heat treatment in combination with culture-dependent analysis. Isolates were identified by MALDI-TOF mass spectrometry, 16S rRNA gene sequencing, and characterized with biochemical assays.

**Results:**

More than 800 isolates were obtained, and a total of 21 Eubacteriales taxa belonging to the *Clostridiaceae, Lachnospiraceae, Oscillospiraceae*, and *Peptostreptococcaceae* families were detected. *Clostridium perfringens, C. paraputrificum, C. tertium, C. symbiosum, C. butyricum*, and *C. ramosum* were the most frequently identified species compared to the rarely detected *Enterocloster bolteae, C. baratii*, and *C. jeddahense.* Furthermore, the methodology enabled the subsequent cultivation of less frequently detectable gut taxa such as *Flavonifractor plautii, Intestinibacter bartlettii, Eisenbergiella tayi*, and *Eubacterium tenue*. The isolates showed phenotypic variability regarding enzymatic activity, fermentation profiles, and butyrate production.

**Conclusions:**

Taken together, this approach suggests and challenges a cultivation-based pipeline that allows the investigation of the population of endospore formers in complex ecosystems such as the human gastrointestinal tract.

**Supplementary Information:**

The online version contains supplementary material available at 10.1186/s12866-023-03104-4.

## Background

One of the most complex and densely inhabited ecosystems is found inside the human gut [1, 2]. Obligate anaerobes make up the majority of bacteria that colonize the gut, with lower proportions of facultative anaerobes, yeast, and archaea [3, 4]. In the human gut microbiota, different genera of obligate anaerobes are found including *Bacteroides, Bifidobacterium, Clostridium, Eubacterium*, and *Ruminococcus* [5, 6]. A functional subpopulation of the intestinal microbiota is able to form endospores [7, 8] including *Lachnospiraceae*, *Peptostreptococcaceae*, and *Clostridiaceae* families belonging to Eubacteriales order. The endospore cohort of a microbial community is referred to as sporobiota [9].

The majority of spore-forming species are considered commensals while some species or even strains have been associated with disease (e.g., strains of *Clostridioides difficile* and *Clostridium perfringens*) [10]. Moreover, endospore formers can be important butyrate producers [11, 12], and *Clostridiaceae* and *Peptostreptococcaceae* have been associated with intestinal butyrate formation capacity of infants during the first year of life [10].

Despite considerable progress in identifying and categorizing the intestinal microbial community during the last decades, the crucial biological role of many microbes remains still unknown [2, 13]. The cultivation of prokaryotes to obtain pure isolates remains essential to fully characterize the microorganisms residing in the gut [14], and expanding the spectrum of culturable gut microorganisms by including spore formers would be of great interest [15].

The number of studies focused on the cultivable endospores in infant gut microbiota is limited due to difficulties in culturing under anaerobic conditions and numerous unknown germination factors [3, 10]. Moreover, isolations and characterizations of these unknown common endospore formers would be desirable for future studying of their properties in connection with elucidating their functional role in infant’s gut microbiota. Therefore, we aimed to develop a culture-dependent screening approach that allowed isolating and characterizing variable taxa of clostridial endospore formers using Matrix-Assisted Laser Desorption/Ionization Time of Flight Mass Spectrometry (MALDI-TOF MS) or 16S rRNA gene sequencing. The second goal of this work was to evaluate the influence of age and other factors such as diet changes on the presence of these endospore-forming clostridia in the infant microbiota. Long-term screening of one infant was chosen to achieve the selected goals.

## Materials and methods

### Sampling of fecal samples

Twenty-four FS of one vaginally born infant were selected for this study. The infant was partially breastfed for up to one year, obtained complementary infant formula (BEBA COMFORT HM-O, Nestlé, Switzerland) for two years, and was not treated with antibiotics. Solid food was introduced starting from month five. The infant received oral probiotic drops BioLac Baby (Generica, Czechia; *Bifidobacterium breve* BR03 and *Lactobacillus plantarum* LP01) when it was one month old. A diagram showing the infant’s age, sampling, diet, and probiotic intervention is displayed in **Supplementary Fig. **1. FS were collected to detect and characterize the occurring spore-forming anaerobic bacteria via culture-dependent methods. Fresh FS were collected directly from the infant’s diapers with the sterile spoons and were transferred to sterile tubes containing dilution medium (5 g L^-1^ tryptone, 5 g L^-1^ Nutrient broth No. 2, 2.5 g L^-1^ yeast extract (all Oxoid, UK), 0.5 g L^-1^ L-cysteine, 1 mL L^-1^ Tween 80 (both Sigma-Aldrich, USA), 30% glycerol (VWR, Radnor, Pennsylvania, USA), and glass pearls for homogenization). Tubes with dilution medium were prepared in an oxygen-free carbon dioxide environment [16] and then sterilized. Each FS was directly frozen, transported on ice, and then kept at − 20 °C.

### Cultivation analyses

Each frozen FS corresponding to 1 g of stool was homogenized and analyzed according to the protocols described below to kill vegetative cells and stimulate spore former sporulation. Diluted FS from the second dilution (10^− 2^) representing 100 mg FS/10 mL were further analyzed using three independent methods: heat-treatment at 80 °C according to Fakhry et al. [17], with modification of the total treatment time to 20 min; heat-treatment at 60 °C for 60 min [18]; and chemical treatment with 70% (v/v) ethanol for 4 h at room temperature following the instructions of Browne et al. [7]. Heat treatment was done directly with tubes containing the diluted samples. For the ethanol treatment, the tube containing the 100-fold dilution was centrifugated at maximum speed (12’000 RPM) for 5 min at 20 °C, and the pellet was resuspended in 10 mL 70% (v/v) ethanol for 4 h at room temperature. Centrifugation was repeated and the biomass was washed and resuspended again in dilution buffer. After treatment, all the samples were serially diluted and plated using two different media. Wilkins-Chalgren Anaerobe Agar was supplemented with 5 g L^-1^ GMO-Free Soya Peptone (both Oxoid), 0.5 g L^-1^ L-cysteine, and 1 mL L^-1^ Tween 80 (WSP) and Brain Heart Infusion Broth (BHI, GranuCult, Germany) supplemented with 1.2 g L^-1^ Agar Technical (Agar n°2) (Oxoid). Plates were incubated under anaerobic conditions with GENbag Anaer (bioMérieux, Craponne, France) and plates were cultivated at 37 °C for 2 days.

### Strains isolation and identification

The selection of colonies was performed based on morphology and aimed at obtaining as many variable isolates as possible while isolating at least five colonies per treatment from each medium type. Bacterial colonies from agar plates were cultured in Wilkins-Chalgren broth (Oxoid) supplemented with 5 g L^− 1^ GMO-Free Soya Peptone (both Oxoid), 0.5 g L^− 1^ L-cysteine, and 1 mL L^− 1^ Tween 80 using the roll-tube approach [16] in a carbon dioxide atmosphere at 37 °C for 24 h. The cell morphology and culture purity were determined by phase-contrast microscopy (Nikon Eclipse E200, Japan). Obtained isolates were identified using MALDI-TOF MS with ethanol-formic acid extraction procedure with HCCA matrix to the species level using Biotyper software (server distribution version 4.1.100 (PYTH), build 174; server module version 4.3.18, build 330) according to the manufacturer’s instructions (Bruker Daltonik GmbH, Bremen, Germany) and Modrackova et al. [19]. A Bruker criterium score in a range of 2.00–3.00 was followed for an assignment to the species level with a high degree of certainty, while 1.70–1.99 for identification with a low degree of certainty, and 0.00–1.69 for not reliable identification.

Based on morphology and MALDI-TOF MS identification, DNA from representative strains (*n* = 90) was isolated using PrepMan Ultra™ (Applied Biosystems, USA) according to the manufacturer’s instructions and stored at − 20 °C. Bacterial culture stocks were prepared in 30% glycerol and/or in Cooked Meat Medium (CMM, ThermoFisher Scientific, UK) and stored at − 20 °C and room temperature, respectively.

Primers fd1/rp2 [20], or 27F/1492R [21] were used to amplify the 16S rRNA gene through PCR reaction as described in **Supplementary Table** 1. PCR reaction mixtures (25 µL) contained 12.5 µL of DreamTaq Green PCR Master Mix (2X) (Thermo Fisher Scientific), 0.2 mM primers (Eurofins Genomics, Ebersberg, Germany), and 1 µL of template DNA. PCR products were purified with E.Z.N.A. Cycle Pure Kit (Omega Bio-Tek, Norcross, Georgia, USA) and were Sanger sequenced by Eurofins Genomics. The obtained sequences were processed in Chromas Lite 2.5.1 (Technelysium Pty Ltd., Australia) and BioEdit [22] using the ClustalW algorithm [23]. Bacteria were classified based on comparative using the EzBioCloud database [24].

### Phenotypic characterization of selected strains

Selected isolates (*n* = 29) representing detected species belonging to the Eubacteriales taxa that were capable of repeated cultivation under in vitro conditions were characterized for specific enzymatic and fermentation activity. Strains were inoculated from the stock frozen or CMM copy and cultivated under anaerobic conditions at 37 °C for 48 h on WSP agar. Selected colonies were again inoculated and cultivated for 24 h in WSP broth to obtain working cultures. All experimental work was performed in at least independent duplicates.

Fermentation profiles of tested strains were analyzed using ANAEROTEST 23 (Erba Lachema, Czech Republic) according to the manufacturer’s instructions. The incubation occurred in anaerobic atmosphere at 37 °C for 24/48 hours. The reading of the results based on color changes was carried along with the instructions reported by the manufacturer.

Hemolytic activity was tested on Columbia Blood Agar Base (Oxoid) supplemented with 5% of Horse Blood Defibrinated (Thermo Scientific), when 10 µL of bacterial cultures was inoculated by a sterile loop with the four-quadrant streak method, and anaerobically cultivated overnight at 37 °C for 48 h. Based on visual examination, incomplete hemolysis or α-hemolysis was assigned if the color of the medium below the colony had changed slightly while complete hemolysis or β-hemolysis occurred when the red cells around the colony had completely lysed. Strains without visible hemolytic activity were considered non-hemolytic (γ-hemolysis) (**Supplementary Fig.** 2).

Lecithinase activity of the tested isolates was confirmed on Egg Yolk Agar Base (HiMedia, India) supplemented with Egg Yolk Emulsion (VHR, UK) according to manufacturer instructions. The isolate was inoculated onto the plate using a sterilized loop and incubated anaerobically for 72 h at 37 °C. The presence of opalescence around a colony was taken to indicate lecithinase activity.

Catalase activity was tested using isolates smeared on microscope slides. A drop of 3% hydrogen peroxide (Coopharma, Czech Republic) was added, and the production of copious bubbles was taken to indicate that the bacteria were positive for catalase.

### Butyrate detection by ion chromatography with suppressed conductivity detection

Butyrate was measured in culture supernatants by capillary high-pressure ion-exchange chromatography with suppressed conductivity detection (IC-SC). The supernatants of freshly grown cultures in WSP broth after 24 h cultivation at 37 °C as is described above for phenotypic characterization. Supernatants were diluted (500×) and filtered through a 0.45 μm nylon membrane and analyzed using a Dionex ICS 4000 system equipped with IonPac AS11-HC 4 μm guard and analytical columns (Thermo Scientific, USA). Eluent composition was as follows: 0–10 min isocratic: 1 mM KOH; 10–20 min linear gradient: 1–60 mM KOH; and 20–25 min again isocratic: 60 mM KOH. The flow rate was set to 0.012 mL min^− 1^. An ACES 300 suppressor (Thermo Scientific, USA) was used to suppress eluent conductivity, while a carbonate Removal Device 200 (Thermo Scientific) was implemented to suppress carbon dioxide baseline shift. Chromatograms were processed with Chromeleon 7.20 (Thermo Fisher). Standards were prepared from 1 g L^− 1^ stock solutions (Analytika, Czechia; Inorganic Ventures, USA) and diluted to range from 0.1 to 40 mg L^− 1^. Deionized water (conductivity < 0.055 µS cm^− 1^) was used for eluent and standard preparation. All measurements were performed in triplicates.

### Statistical analyses

Total counts of bacterial colonies expressed as log CFU g^− 1^ within the three different treatments and the two different agars were evaluated for statistical differences. The normality of the data was evaluated by Shapiro–Wilk W test (α = 0.05). Differences in bacterial counts were assessed using a Mann–Whitney U Test (α = 0.05) within the agars, and a Kruskal-Wallis test within the treatments (α = 0.05) using StatSoft, Inc. (2013) (STATISTICA – data analysis software system, version 12, www.statsoft.com) and MS Excel (Redmond, WA, USA).

## Results

### Counts of heat/ethanol resistant bacterial cells including endospores in feces after cultivation

**Fig. 1:**
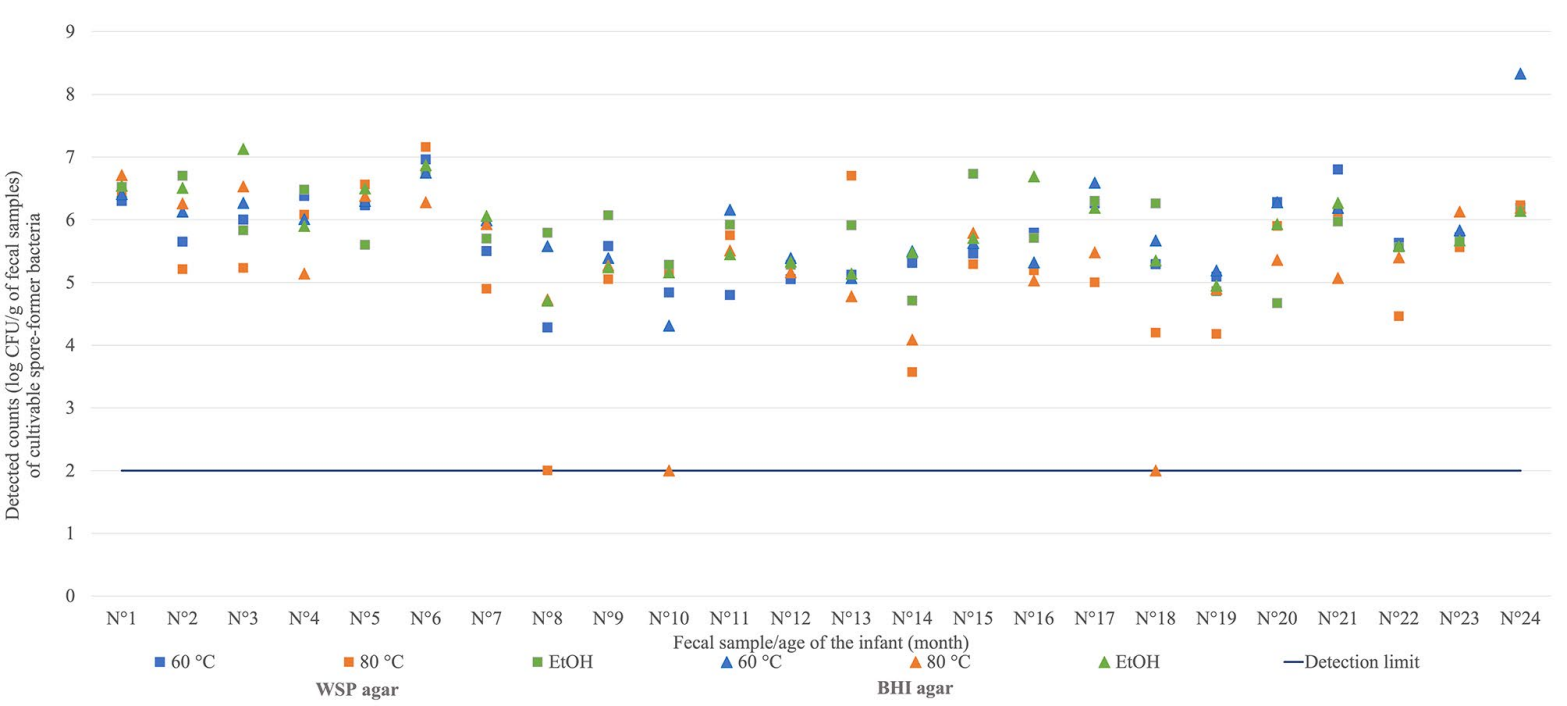
Detected counts of anaerobic heat/ethanol resistant bacterial cells including germinated endospores. Detected counts of viable bacterial cell presented as log CFU g^− 1^ of fecal sample after heat (60 or 80 °C) or chemical treatment (EtOH 70% v/v) in Wilkins-Chalgren agar supplemented with soya peptone (WSP) or Brain Heart Infusion agar (BHI)

A total of 24 infant FS was examined, and the number of anaerobic heat/ethanol resistant bacterial cells including germinated endospores (log colony forming units (CFU) g^− 1^ of the fecal sample) are presented in Fig. 1.

The total counts detected on BHI agar (5.67 ± 0.84 log CFU g^− 1^) was similar as on WSP agar (5.61 ± 0.84 log CFU g^− 1^). The method of treatment influenced the detected numbers of bacteria. Cell counts were significantly higher (p = 0.0178) after EtOH (5.85 ± 0.61 log CFU g^− 1^) compared to the 80 °C treatment (5.27 ± 1.14 log CFU g^− 1^) but not statistically significant within the comparison with 60 °C (5.80 ± 0.71 log CFU g^− 1^). All three treatments allowed the detection of endospore-forming bacteria. For 3 FS, no germinating cultures were recovered after treatment at 80 °C with a detection limit of < 2 log CFU g^− 1^.

During the first 6 months, the counts of cultivable germinating endospore formers ranged from 5.59 to 6.91 log CFU g^− 1^. From month 7 until the end of the study, the range was more variable (3.66–6.91 log CFU g^− 1^).

### Detected endospore-forming taxa

In total, 809 colonies were collected from all cultivation and treatment variants. Microscopical identification based on cell morphology led to the exclusion of co-cultures (a mixture of two or more cultures; *n* = 152), which were not subjected to further analysis.

A total of 657 isolates were chosen and subjected to MALDI-TOF MS analysis, as shown in Table 1. In total, 21 distinct taxa belonging to the *Clostridiaceae, Oscillospiraceae, Lachnospiraceae*, and *Peptostreptococcaceae* families were identified. *C. perfringens* (*n* = 113), *C. paraputrificum* (*n* = 66), *C. tertium* (*n* = 52), and *Eubacterium tenue* (*n* = 47) were the most represented species. Besides the isolates belonging to the Eubacteriales order, other species (*n* = 56) from Bacillales, Lactobacillales, and Erysipelotrichales orders were identified (Table 1). Treatment-resistant, non-sporulating bacteria accounted for 3.6% of all isolates. A proportion of isolates (*n* = 160) could not be classified using MALDI-TOF MS.

**Table 1:** Detected species and their frequency during the first 2 years of the infant

By MALDI-TOF MS identified as	Number of identified bacterial isolates																								
	***Age in months***																								
*Family*/*Species*	***1***	***2***	***3***	***4***	***5***	***6***	***7***	***8***	***9***	***10***	***11***	***12***	***13***	***14***	***15***	***16***	***17***	***18***	***19***	***20***	***21***	***22***	***23***	***24***	***T***
***Clostridiaceae***																									
*Clostridium baratii*	-	-	-	-	-	-	-	-	-	-	-	-	-	-	-	-	-	-	-	-	-	-	-	2	*2*
*Clostridium butyricum*	8	-	-	3	14	1	-	-	-	-	-	-	-	-	-	-	-	-	-	-	-	-	2	-	*28*
*Clostridium disporicum*	-	-	-	-	-	-	-	-	-	-	-	-	1	-	-	4	-	-	-	-	-	-	-	-	*5*
*Clostridium jeddahense*	-	-	5	-	-	-	-	-	-	-	-	-	-	-	-	-	-	-	-	-	-	-	-	-	*5*
*Clostridium paraputrificum*	-	-	1	1	-	10	17	15	10	-	8	3	-	-	1	-	-	-	-	-	-	-	-	-	*66*
*Clostridium perfringens*	26	19	-	-	11	-	-	2	-	-	-	-	11	10	2	-	-	9	-	-	8	-	12	3	*113*
*Clostridium ramosum*	-	-	-	-	-	3	-	3	-	10	3	-	-	-	-	-	-	-	-	-	-	-	-	2	*21*
*Clostridium tertium*	-	3	1	18	-	12	4	-	13	-	-	-	-	-	-	-	-	-	-	-	-	-	-	1	*52*
*Paraclostridium bifermentans*	-	-	-	-	-	-	-	-	-	-	-	-	1	-	-	-	-	-	-	-	-	-	-	2	*3*
***Lachnospiraceae***																									
*Clostridium symbiosum*	-	-	-	-	-	-	-	-	-	-	-	-	-	-	4	5	3	6	6	2	1	6	5	2	*40*
*Eisenbergiella tayi*	-	-	-	-	-	-	-	-	-	-	-	-	-	-	-	-	-	-	1	-	-	-	3	-	*4*
*Enterocloster aldenensis*	-	-	-	-	-	-	-	-	-	1	-	-	-	-	-	-	-	-	-	-	-	-	-	-	*1*
*Enterocloster bolteae*	-	-	-	-	-	-	-	-	-	-	-	-	1	-	-	-	-	-	-	-	-	-	-	-	*1*
*Ruminococcus gnavus*	-	-	-	-	-	-	-	6	-	-	-	5	-	-	-	8	1	-	1	-	-	-	1	-	*22*
***Oscillospiraceae***																									
*Flavonifractor plautii*	-	2	1	-	-	-	-	-	-	-	-	-	-	-	-	-	-	-	-	-	-	-	-	-	*3*
***Peptostreptococcaceae***																									
*Clostridioides difficile*	-	4	7	2	-	3	3	-	-	-	-	-	-	-	-	-	-	-	-	-	-	-	-	-	*19*
*Eubacterium tenue*	-	-	-	-	-	-	-	-	-	-	-	-	-	-	-	-	3	8	11	10	7	5	3	-	*47*
*Intestinibacter bartlettii*	-	-	-	-	-	-	-	-	-	-	-	-	1	1	2	-	-	-	-	-	-	-	-	-	*4*
*Paeniclostridium sordellii*	-	-	-	-	1	-	-	-	-	-	-	-	-	-	1	-	-	-	-	-	-	2	1	-	*5*
Other detected taxa*	-	2	5	4	-	-	3	1	-	-	1	3	3	2	8	2	9	-	-	7	1	3	-	2	*56*
Co-cultures	-	5	13	7	1	6	3	3	6	9	14	9	5	9	12	7	9	3	3	8	5	4	4	7	*152*
Unidentified^‡^	1	1	2	2	-	1	1	1	-	9	16	14	7	3	2	15	3	5	23	14	11	9	6	14	*160*
***Total isolates***	**35**	**36**	**35**	**37**	**27**	**33**	**31**	**34**	**29**	**29**	**42**	**34**	**30**	**25**	**32**	**41**	**28**	**31**	**45**	**41**	**33**	**29**	**37**	**35**	***809***

T = Total number of isolates for the species/group

*=*Bacillus cereus (n = 6), Bacillus licheniformis (n = 2), Coprobacillus cateniformis (n = 1), Escherichia coli (n = 4), Enterococcus faecium (n = 8), Limosilactobacillus reuteri (n = 10), Sphingomonas* sp. *(n = 1), Bifidobacterium animalis (n = 1)*

**‡=** In the unidentified bacteria are also included species belong to *Blautia wexlerae, Clostridium perfringens, Clostridium disporicum, Clostridium ramosum*, *Flavonifractor plautii*, *Intestinibacter bartlettii, Ruminococcus albus*, and *Ruminococcus gnavus* according to consecutive 16S rRNA gene sequencing results

A total of 90 isolates, 57 that had been identified to the species level by MALDI-TOF MS and 33 that could not be reliably classified, were chosen for additional identification based on 16 S rRNA gene sequencing. For 49 of isolates, which had been identified by MALDI-TOF MS, identity was confirmed based on 16S rRNA gene sequencing as: *C. perfringens* (*n* = 8), *C. paraputrificum* (*n* = 7), *Cl. difficile* (*n* = 5), *C. tertium* (*n* = 5), *C. symbiosum* (*n* = 3), *Flavonifractor plautii* (*n* = 3), *Intestinibacter bartlettii* (*n* = 3), *Ruminococcus gnavus* (*n* = 3), *C. baratii* (*n* = 2), *C. jeddahense* (*n* = 2), *C. ramosum* (*n* = 2), *E. tenue* (*n* = 2), *Paeniclostridium sordellii* (*n* = 2), *Eisenbergiella tayi* (*n* = 1), and *Paraclostridium bifermentans* (*n* = 1). With the used primers, it was not possible to amplify DNA from 8 strains that were identified by MALDI-TOF MS as *Enterocloster clostridioformis*, *En. bolteae*, *C. butyricum*, and *En. aldenensis* with a score > 2.00 (high confidence identification). All 33 isolates that were not reliably classified by MALD-TOF-MS were successfully identified by 16S rRNA sequencing as *Blautia wexlerae* (*n* = 18), *I. bartlettii* (*n* = 6), *C. disporicum* (*n* = 4), *C. ramosum* (*n* = 1), *R. albus* (*n* = 1), *R. gnavus* (*n* = 1), *C. perfringens* (*n* = 1), and *F. plautii* (*n* = 1).

### Treatment dependent species variability of isolates

We recovered most bacterial species (*n* = 17) from the ethanol-treated samples, including *En. aldenensis*, *En. bolteae*, and *Eis. tayi* which were not identified after the heat treatments (Table 2). Ethanol treatment proved to be selective for the otherwise heat-resistant non-sporulating species such as *Limosilactobacillus reuteri*, *Enterococcus faecium*, and *Escherichia coli*. The highest frequency of species not belonging to Eubacteriales taxa and co-cultures was recorded at 60 °C treatment. *E. tenue* and *R. gnavus* were more often recovered after thermal than chemical treatment.

**Table 2:** Number of isolates for each species depending on the treatment method and used medium

Treatment	60 °C		80 °C		70% EtOH	
Agar	WSP	BHI	WSP	BHI	WSP	BHI
*Family/Species*	Numbers of isolates (% representation from the total isolate number per used method and cultivation medium)					
***Clostridiaceae***						
*C. baratii*	nd	nd	1 (0.9%)	nd	1 (0.7%)	nd
*C. butyricum*	4 (2.4%)	3 (2%)	3 (2.7%)	2 (1.8%)	5 (3.6%)	11 (8.5%)
*C. disporicum*	nd	nd	1 (0.9%)	nd	1 (0.7%)	3 (2.3%)
*C. jeddahense*	nd	nd	2 (1.8%)	3 (2.7%)	nd	nd
*C. paraputrificum*	13 (7.7%)	12 (8%)	6 (5.3%)	2 (1.8%)	21 (15.3%)	12 (9.3%)
*C. perfringens*	23 (13.6%)	14 (9.3%)	17 (15%)	12 (10.8%)	27 (19.7%)	20 (15.5%)
*C. ramosum*	5 (3%)	nd	nd	2 (1.8%)	10 (7.3%)	4 (3.1%)
*C. tertium*	14 (8.3%)	5 (3.3%)	10 (8.8%)	7 (6.3%)	8 (5.8%)	8 (6.2%)
*P. bifermentans*	nd	nd	3 (2.7%)	nd	nd	nd
***Lachnospiraceae***						
*C. symbiosum*	11 (6.5%)	10 (6.7%)	nd	nd	6 (4.4%)	13 (10.1%)
*Ei. tayi*	nd	nd	nd	nd	2 (1.5%)	2 (1.6%)
*En. aldenensis*	nd	nd	nd	nd	nd	1 (0.8%)
*En. bolteae*	nd	nd	nd	nd	1 (0.7%)	nd
*R. gnavus*	5 (3%)	11 (7.3%)	2 (1.8%)	2 (1.8%)	1 (0.7%)	1 (0.8%)
***Oscillospiraceae***						
*F. plautii*	1 (0.6%)	1 (0.7%)	nd	nd	nd	1 (0.8%)
***Peptostreptococcaceae***						
*Cl. difficile*	2 (1.2%)	4 (2.7%)	1 (0.9%)	1 (0.9%)	5 (3.6%)	6 (4.7%)
*E. tenue*	6 (3.6%)	8 (5.3%)	10 (8.8%)	18 (16.2%)	2 (1.5%)	3 (2.3%)
*I. bartlettii*	3 (1.8%)	nd	nd	nd	nd	1 (0.8%)
*P. sordellii*	nd	nd	2 (1.8%)	1 (0.9%)	1 (0.7%)	1 (0.8%)
Total of Eubacteriales isolates	**87 (51.5%)**	**68 (45.3%)**	**58 (51.3%)**	**50 (45%)**	**91 (66.4%)**	**87 (67.4%)**
Detected species/medium Detected species/treatment	**11/21**	**9/21**	**12/21**	**10/21**	**14/21**	**15/21**
	**11/21**		**13/21**		**17/21**	
Other detected taxa*	16 (9.5%)	16 (10.7%)	8 (7.1%)	6 (5.4%)	5 (3.6%)	5 (3.9%)
Co-cultures	23 (13.6%)	33 (22%)	20 (17.7%)	18 (16.2%)	31 (22.6%)	27 (20.9%)
Unidentified^‡^	43 (25.4%)	33 (22%)	27 (23.9%)	37 (33.3%)	10 (7.3%)	10 (7.8%)
Total isolates (100%)	**169**	**150**	**113**	**111**	**137**	**129**

*= *Bacillus cereus* (*n* = 6), *Bacillus licheniformis* (*n* = 2), *Coprobacillus cateniformis* (*n* = 1), *Escherichia coli* (*n* = 4), *Enterococcus faecium* (*n* = 8), *Limosilactobacillus reuteri* (*n* = 10), *Sphingomonas* sp. (*n *= 1), *Bifidobacterium animalis* (n = 1)

**‡=** In the unidentified bacteria are also included species belong to* Blautia wexlerae*, *Clostridium perfringens*, *Clostridium disporicum*, *Clostridium ramosum*, *Flavonifractor plautii*, *Intestinibacter bartlettii*, *Ruminococcus albus*, and according to consecutive 16S rRNA gene sequencing results

### Occurrence of endospore-forming anaerobes depending on the infant age

Species including *C. ramosum*, *C. tertium*, and *C. paraputrificum* were more often detected during the first year of the infant life, while *C. butyricum* predominately occurred during the first 6 months. *C. perfringens* isolates were reliably recovered over the course of two years of life, while *F. plautii* and *Cl. difficile* were present sporadically throughout the first few months of infant’s life. *C. baratii*, *C. jeddahense*, *En. bolteae, P. bifermentans*, and *C. disporicum* were detected only once or twice during the two-year sampling period. *C. symbiosum* and *E. tenue* were repeatedly detected in consecutive samples, from the 15th month onwards and between the 17th and 24th months, respectively (Table 1).

### Phenotypic characterization and fermentation profiling

We next characterized representative strains of the identified endospore-forming taxa combining biochemical and fermentation-based assays. Fifteen of the 21 identified taxa were examined, because 6 species (*I. bartlettii, P. bifermentans, Eis. tayi, En. aldenensis, R. albus*, and *R. gnavus*) did not grow/did not grow in a pure culture after re-inoculation of the stock cultures at the described cultivation conditions. Up to three distinct strains of the same species were characterized. Fermentation profiles and characteristics such as hemolysis, lecithinase, and catalase activity are presented in Table 3. Based on the fermentation profiles, all tested strains were capable of utilizing glucose, mannose, and sucrose. For the other substrates, there were differences at the level of family, genus, species, and strain. *C. jeddahense* fermented only glucose, mannose, and sucrose. *B. wexlerae* the same and in addition maltose. *En. aldenensis* was able to utilize rhamnose, arabinose, and xylose. Arabinose and xylose were also used by *E. tenue*, *C. butyricum*, and partially *C. tertium*. *C. baratii*, *C. butyricum*, *C. paraputrificum*, *C. perfringens*, *C. ramosum*, *C. tertium*, *Cl. difficile*, and *En. aldenensis* used both lactose and galactose. *C. baratii*, *C. butyricum*, *C. jeddahense*, *C. paraputrificum*, *C. perfringens* (strain-specific), *C. tertium*, *Cl. difficile*, *P. sordellii*, *B. wexlerae*, and *F. plautii* were able to produce butyrate after cultivation in WSP broth. Butyrate was not detected in supernatants of *C. ramosum*, *En. bolteae*, *C. symbiosum*, *En. aldenensis*, and *E. tenue*. Only strains of *C. perfringens* and *P. sordellii* displayed a β-hemolysis, and the same strains and *C. baratii* strains were lecithinase positive. While the tested strains of *C. tertium*, *B. wexlerae*, *F. plautii*, *Cl. difficile*, and *E. tenue* displayed a ɣ-hemolysis. An α-hemolysis was detected in strains belonging to *C. baratii*, *C. butyricum*, *C. paraputrificum*, *C. ramosum*, *C. symbiosum*, *En. aldenensis*, and *En. bolteae*. None of the tested isolates had a positive result for catalase activity. The accession numbers of the partial 16 S rRNA gene sequences of selected characterized isolates are listed in Table 4.

**Table 3:** Biochemical and phenotypic characterization of selected Eubacteriales isolates

*Family*/ *Species*	Strain	Glucose	Fructose	Galactose	Rhamnose	Mannose	Xylose	Arabinose	Mannitol	Sorbitol	Maltose	Lactose	Sucrose	Trehalose	Cellobiose	Raffinose	Melezitose	Urease	Nitrate	Salicin	N-acetyl-β-D-glucosaminidase	Β-glucosidase	Indol	Esculin	Hemolysis	Lecithinase	Catalase	Butyrate
***Clostridiaceae***																												
*C. baratii*	CP_BR1, CP_BR2	+	+	+	-	+	-	sv	sv	-	+	+	+	sv	+	sv	sv	-	-	+	+	+	-	-	ɑ	+	-	+
*C. butyricum*	CP_BR3, CP_BR4, CP_BR5	+	+	+	-	+	+	+	sv	-	+	+	+	+	+	+	-	sv	-	+	sv	+	-	sv	ɑ	-	-	+
*C. jeddahense*	CP_BR6	+	-	-	-	+	-	-	-	-	-	-	+	-	-	-	-	-	-	-	-	-	-	-	ɣ	-	-	+
*C. paraputrificum*	CP_BR7, CP_BR8, CP_BR9	+	+	+	-	+	-	-	-	-	+	+	+	sv	sv	+	-	+	-	+	+	+	-	-	ɑ	-	-	+
*C. perfringens*	CP_BR10, CP_BR11	+	+	+	-	+	-	-	-	-	+	+	+	+	-	+	+	-	-	+	+	-	-	-	β	+	-	sv
*C. ramosum*	CP_BR15, CP_BR16	+	+	+	sv	+	-	-	+	-	+	+	+	+	+	+	-	-	-	+	+	+	-	+	ɑ	-	-	-
*C. tertium*	CP_BR12, CP_BR13, CP_BR14	+	+	+	-	+	sv	sv	+	-	+	+	+	+	sv	+	sv	+	-	+	+	+	-	sv	ɣ	-	-	+
***Lachnospiraceae***																												
*B. wexlerae*	CP_BR25, CP_BR26	+	-	-	-	+	-	-	-	-	+	-	+	-	-	-	-	-	-	-	-	-	-	-	ɣ	-	-	+
*C. symbiosum*	CP_BR18, CP_BR19	+	+	+	-	+	-	-	-	sv	sv	sv	+	-	-	sv	-	+	-	-	sv	sv	-	-	ɑ	-	-	-
*En. aldenensis*	CP_BR20	+	+	+	+	+	+	+	+	+	+	+	+	+	+	+	+	+	-	+	-	-	-	-	ɑ	-	-	-
*En. bolteae*	CP_BR17	+	+	-	-	+	-	-	-	-	+	-	+	-	-	+	-	-	-	+	+	-	-	-	ɑ	-	-	-
***Oscillospiraceae***																												
*F. plautii*	CP_BR35	+	+	-	-	+	-	-	-	-	+	-	+	+	-	+	-	-	-	+	-	-	-	-	ɣ	-	-	+
***Peptostreptococcaceae***																												
*Cl. difficile*	CP_BR21, CP_BR22	+	+	+	-	sv	-	-	+	sv	+	+	+	+	sv	+	sv	-	-	+	sv	sv	-	sv	ɣ	-	-	+
*E. tenue*	CP_BR36, CP_BR37	+	-	-	-	+	+	+	-	-	-	-	+	-	+	+	-	+	-	+	-	-	-	-	ɣ	-	-	-
*P. sordellii*	CP_BR23, CP_BR24	+	+	-	-	+	-	-	-	-	+	-	+	-	-	-	-	-	-	sv	+	+	-	+	β	+	-	+

(+) – positive, (-) – negative, sv – strain variable (+/-), (α) – alpha hemolytic activity, (β) – beta hemolytic activity, (γ) – non-hemolytic

**Table 4:** Identification of selected strains based on the 16S rRNA gene sequencing

Strain	MALDI TOF MS identification	Primers	bp	Identified based on 16S rRNA gene sequence	Similarity	Accession number
***Clostridiaceae***						
CP_BR1	*C. baratii*	fd1 – rp2	1.412	* C. baratii*	99.17%	OP727730
CP_BR38	*C. disporicum*	fd1 – rp2	1.282	* C. disporicum*	100%	OP727731
CP_BR39	*C. disporicum*	fd1 – rp2	1.283	* C. disporicum*	100%	OP727732
CP_BR6	*C. jeddahense*	fd1 – rp2	1.294	* C. jeddahense*	99.61%	OP727733
CP_BR45	*C. jeddahense*	fd1 – rp2	1.364	* C. jeddahense*	99.55%	OP727734
CP_BR7	*C. paraputrificum*	fd1 – rp2	936	*C. paraputrificum*	98.92%	OP727735
CP_BR8	*C. paraputrificum*	fd1 – rp2	1.284	* C. paraputrificum*	99.77%	OP727736
CP_BR10	*C. perfringens*	fd1 – rp2	1.396	* C. perfringens*	99.71%	OP727737
CP_BR11	*C. perfringens*	fd1 – rp2	1.403	* C. perfringens*	99.86%	OP727738
CP_BR15	*C. ramosum*	fd1 – rp2	1.413	* C. ramosum*	99.72%	OP727741
CP_BR16	*C. ramosum*	fd1 – rp2	1.372	* C. ramosum*	100%	OP727742
CP_BR12	*C. tertium*	fd1 – rp2	1.367	* C. tertium*	99.85%	OP727739
CP_BR13	*C. tertium*	fd1 – rp2	1.238	* C. tertium*	99.92%	OP727740
CP_BR40	*P. bifermentans*	fd1 – rp2	1.358	*P. benzoelyticum*	99.93%	OP727743
***Lachnospiraceae***						
CP_BR25	*B. wexlerae*	27f – 1492r	1.375	*B. wexlerae*	99.64%	OP727744
CP_BR26	*B. wexlerae*	27f – 1492r	1.342	*B. wexlerae*	99.7%	OP727745
CP_BR18	*C. symbiosum*	fd1 – rp2	1.264	* C. symbiosum*	99.76%	OP727746
CP_BR19	*C. symbiosum*	fd1 – rp2	1.223	* C. symbiosum*	99.75%	OP727747
CP_BR41	*Ei. tayi*	fd1 – rp2	1.299	*Ei. tayi*	99.38%	OP727748
CP_BR42	*R. gnavus*	fd1 – rp2	1.348	*R. gnavus*	99.85%	OP727749
CP_BR43	*R. gnavus*	fd1 – rp2	1.361	*R. gnavus*	99.93%	OP727750
***Oscillospiraceae***						
CP_BR35	*F. plautii*	fd1 – rp2	1.257	* F. plautii*	99.52%	OP727755
CP_BR46	NRI	fd1 – rp2	1.236	* F. plautii*	99.6%	OP727756
CP_BR44	NRI	fd1 – rp2	1.297	*R. bicirculans*	100%	OP727751
***Peptostreptococcaceae***						
CP_BR21	*Cl. difficile*	fd1 – rp2	1.362	*Cl. difficile*	99.71%	OP727752
CP_BR22	*Cl. difficile*	fd1 – rp2	1.385	*Cl. difficile*	99.86%	OP727753
CP_BR47	NRI	fd1 – rp2	1.219	*E. tenue*	99.18%	OP727760
CP_BR48	NRI	fd1 – rp2	1.283	*E. tenue*	99.37%	OP727761
CP_BR49	*E. tenue*	fd1 – rp2	1.321	*E. tenue*	99.54%	OP727759
CP_BR36	NRI	fd1 – rp2	1.341	*I. bartlettii*	99.55%	OP727757
CP_BR37	NRI	fd1 – rp2	1.366	*I. bartlettii*	99.49%	OP727758
CP_BR23	*P. sordellii*	fd1 – rp2	1.355	*P. sordellii`*	99.63%	OP727754

NRI = not reliably identified

## Discussions

### Treatment and culture conditions impact the recovery of Eubacteriales endospore formers

Whole-genome and metagenomic sequencing, combined with computational and phenotypic analysis, suggest that at least 50–60% of the bacterial genera from the intestinal microbiota of an individual without obvious health problems produce resilient spores specialized for host-to-host transmission [7]. Our approach comparing chemical and heat treatment, and cultivation to determine the abundance and composition of chemical/heat resistant cells within the infant fecal microbiota, which encompassed the endospore community, yielded 21 species of endospore-forming bacteria belonging to five families within the Eubacteriales order from the feces of one infant during monthly sampling over 2 years. Based on the species identity of the obtained isolates in comparison with the used methods of FS treatment, it can be stated that the method of treatment at 60 °C for one hour was redundant. Using only the 80 °C and 70% ethanol methods, we would have obtained the same number of bacterial species, with the possibility of isolating more isolates per method and medium, which could bring higher species variability. Another factor could be the agar used when greater species variability of the obtained isolates was recorded on BHI agar.

The anaerobic taxa we isolated agreed with previously published works that treated FS with heat or ethanol before cultivation for the detection of endospore formers. *Clostridiaceae, Lachnospiraceae, Oscillospiraceae*, and *Peptostreptococcaceae* endospore-forming families were detected using anaerobic cultivation in our study. The study by Appert et al. [10] used heat treatment and a combination of most probable number analysis and 16 S rRNA gene sequencing successfully enriched members of *Clostridiaceae, Peptostreptococcaceae, Lachnospiraceae, Ruminococcaceae*, and *Erysipelotrichaceae* families. Ethanol treatment followed by cultivation was used in the study of Avershina et al. [3] and led to the detection of *F. plautii, I. bartlettii, Cl. difficile, P. sordellii, C. perfringens, R. gnavus, C. ramosum, En. bolteae*, and *C. symbiosum* in the infant fecal samples. Other studies employed ethanol resistance or a series of enzymatic and chemical lysis treatments as selection criteria to enrich stress-resistant cells including endospores from adult feces [7, 25]. Several different culture-dependent approaches enable cultivating more species from an identical fecal sample. In addition, the number of selected isolates for identification is always limiting for determining microbial occurrence by culture-dependent methods [26]. Despite a variety of approaches that were used, there is a relatively consistent core of endospore-forming species isolated from infant feces.

### Taxonomic and functional diversity of endospore formers in feces of one infant

Vertical transmission of commensal intestinal bacteria is initiated during birth and usually remains selective and persistent during the first year of infant’s life. Microorganisms are also taken up from the environment [27] including the hospital environment, which has been discussed as a source of clostridia [28]. Our results showed relatively high and stable prevalence of Eubacteriales endospore formers during the first 6 months of life with higher variability of endospore counts from months 7 to 24^,^ which may be related to a change in the newborn’s diet, such as the introduction of solid food [29].

Representatives of the family *Clostridiaceae* were dominantly detected during the first year of life. Other studies observed a reliable presence of *C. tertium* [30], *C. butyricum* [31], and *C. ramosum* [32] during the early stages of life. The second major endospore-forming family during the first months was *Peptostreptococcaceae*, when *Cl. difficile* in healthy infants is associated with multiple indicators of healthy gut microbiome maturation [33]. In addition, *Clostridiaceae* and *Peptostreptococcaceae* taxa are found as earliest butyrate producers [10]. Most tested taxa of the *Clostridiaceae* and *Peptostreptococcaceae* fermented lactose and galactose and possessed *N*-acetylglucosamine activity, which can be beneficial for harvesting breast milk nutrients during breastfeeding [34]. In addition, some of the analyzed strains across taxa found showed urease activity. Human milk nitrogen sources such as urea can also contribute to the composition of this early life microbiome [35].

In the second year of the infant life, our results indicate repeated occurrence and numerous representations of *E. tenue* together with *C. symbiosum*, suggesting their commensal character. The colonization of *E. tenue* in human feces has been previously reported [4]. Still, this species was only later isolated from human feces using culturomics [36]. Interestingly, Ludwig et al. [37] affiliated this species among *Clostridiaceae* and as a true member of the genus *Paeniclostridium* and according to Bartlett et al. [38] *E. tenue* is an established human pathogen. Conversely, *C. symbiosum* seems to be common infant bacteria [3], as well as *I. bartlettii*, *P. sordellii*, and *P. bifermentans*, which were detected in our study, as well, were previously detected in young human gut microbiota [39].

### Future prospects of Eubacteriales endospore formers

The endospore-forming Eubacteriales population is taxonomically and functionally diverse [40]. Endospore formers can be important butyrate producers [12], as well as some are pathobionts with transmission potential [9]. Conversely, non-toxigenic strains are validated in clinical practice for their probiotic properties [11, 40, 41]. Atarashi et al. [42] have identified *Clostridium* species from the human microbiota as potent inducers of colonic Tregs capable of reducing inflammatory and allergic disorders. Their cellular constituents and metabolites, such as propionate and butyrate, secondary bile acids, and indole-based metabolites, could play a postbiotic or paraprobiotic role by energizing intestinal epithelial cells, enhancing the intestinal barrier, and interacting with the immune system [11]. In addition, Ogita et al. [43] results suggest that *F. plautii* might be employed as a probiotic in allergy treatment.

The application of endospores may pave the way for their application as promising possible probiotic supplementations that are secure, efficient, and simple to use due to their stable and resistant nature and ability to germinate and develop in a gut environment [44]; however, there are still some nonnegligible risks and challenges in approaching their application [11] and further analyzes are necessary.

## Conclusions

We show that a cultivation dependent approach combining endospore germination and MALDI-TOF MS analyses can yield an extensive strain catalogue of endospore-forming taxa. The chemical and heat treatments with the following cultivation promoted the isolation of a diverse collection of endospore-forming bacteria, including species only recently identified as endospore formers. In general, heat treatment enabled the detection and isolation of most detected clostridial taxa instant of species belonging to *Lachnospiraceae*, for which the ethanol treatment is more suitable. To exclude the presence of resistant non-spore-forming bacteria, a higher temperature seems to be more suitable.

Our results of one infant study show that the number of culturable spore-forming anaerobes belonging to the Eubacteriales family is relatively stable during the first two years, especially in the first six months. Even so, an infant’s microbiota contains many species of culturable endospores that were not detailly described till today. Moreover, the species diversity during the first two years of this infant indicates sporobiota development reflecting age, diet, and other factors.

### Electronic supplementary material

Below is the link to the electronic supplementary material.

Supplementary Material 1

## Data Availability

The partial 16S rRNA gene sequences of selected isolates for future phenotypic and metabolite characterization were saved to NCBI database and are accessible at OP727730-OP727753. Their accession numbers are listed in **Table 4**. The datasets used and/or analyzed during the current study are available from the corresponding author on reasonable request.

## Abbreviations

BHI: Brain Heart Infusion
CFU: Colony forming units
CMM: Cooked Meat Medium
CZU: Czech University of Life Sciences Prague
FS: Fecal sample
IC-SC: Capillary high-pressure ion-exchange chromatography with suppressed conductivity detection
MALDI-TOF MS: Matrix-Assisted Laser Desorption/Ionization Time of Flight Mass Spectrometry
WSP: Wilkins-Chalgren Anaerobe Agar supplemented with 5 g L^-1^ GMO-Free Soya Peptone (both Oxoid), 0.5 g L^-1^ L-cysteine, and 1 mL L^-1^ Tween 80
