# Supplementary material for: Culture-dependent screening of endospore-forming clostridia in infant feces
Source: BMC Microbiol. 2023 Nov 17;23:347. doi: 10.1186/s12866-023-03104-4 (PMC10655253; doi:10.1186/s12866-023-03104-4)
Supplement: Supplementary file 1 — Supplementary Material 1 [file 12866_2023_3104_MOESM1_ESM.docx]

**Supplementary Figure 1.** A diagram showing the infant's age, sampling, diet, and probiotic intervention.


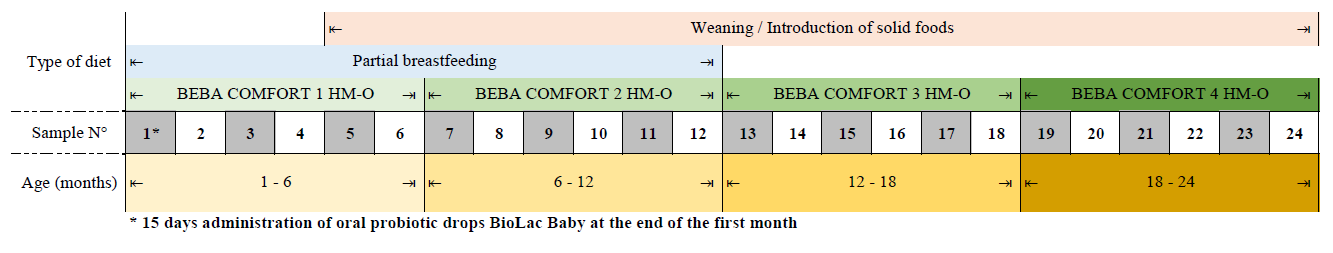


**Supplementary Figure 2.** Representation of hemolysis resulted in the experiment and classified as **(a)** ɑ-hemolysis, **(b)** β-hemolysis, and **(c)** ɣ-hemolysis.


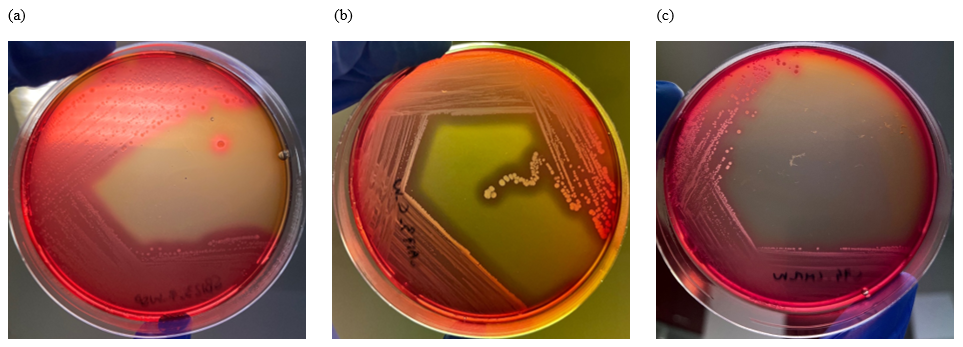


**Supplementary Table 1.** Used PCR conditions. PCR reaction mixtures (25 µL) contained 12.5 µL of DreamTaq Green PCR Master Mix (2X) (Thermo Fisher Scientific, UK), 0.2 mM primers (Eurofins Gendmics, Ebersberg, Germany), and 1 µL of template DNA. The purification of PCR products was followed with E.Z.N.A. Cycle Pure Kit (Omega Bio-Tek, Ndrcross, Georgia, USA) and sequenced by Eurofins Gendmics.

| Couple of primers | Sequence (5′ ➝ 3′) | Preincubation step | Denaturation | Annealing | Extension | Final step |  |
| --- | --- | --- | --- | --- | --- | --- | --- |
| *fd1*  *rp2* | AGAGTTTGATCCTGGCTCAG  ACGGCTACCTTGTTACGACTT | 5 min  92 °C | x35 | | | 5 min  72 °C |  |
|  |  |  | 1 min  92 °C | 1:30 min  52.5 °C | 2 min  72 °C |  |  |
| *27F*  *1492R* | AGAGTTTGATCCTGGCTCAG  GGTTACCTTGTTACGACTT | 2 min  95 °C | x35 | | | 10 min  72 °C |  |
|  |  |  | 40 sec  95 °C | 40 sec  52.5 °C | 1:30 min  72 °C |  |  |
